# Research on Robot Collision Response Based on Human–Robot Collaboration

**DOI:** 10.3390/s26020495

**Published:** 2026-01-12

**Authors:** Sicheng Zhong, Chaoyang Xu, Guoqiang Chen, Yanghuan Xu, Zhijun Wang

**Affiliations:** 1 College of Mechanical Engineering, North China University of Science and Technology, Tangshan 063210, China; 2 Hebei Industrial Robot Research Institute, Tangshan 063210, China

**Keywords:** collision response, human-robot collaboration, adaptive conductance control, collision corresponding algorithms

## Abstract

With the rapid advancement of science and technology, robotics is evolving towards more profound and extensive applications. Nevertheless, the inherent limitations of traditional industrial “caged” robots have significantly impeded the full utilization of their capabilities. Consequently, breaking free from these constraints and realizing human–robot collaboration has emerged as a new developmental trend in the robotics field. The collision-response mechanism, as a crucial safeguard for human–robot collaboration safety, has become a pivotal issue in enhancing the performance of human–robot interaction. To address this, an adaptive admittance control collision-response algorithm is proposed in this paper, grounded in the principle of admittance control. A collision simulation model of the AUBO-i5 collaborative robot is constructed. The effectiveness of the proposed algorithm is verified through simulation experiments focusing on both the end-effector collision and body collision of the robot, and by comparing it with existing admittance control algorithms. Furthermore, a collision-response experimental platform is established based on the AUBO-i5 collaborative robot. Experimental studies on end-effector and body collisions are conducted, providing practical validation of the reliability and utility of the proposed adaptive admittance control collision-response algorithm.

## 1. Introduction

Collaborative robots are widely used in agriculture [1,2,3], industry [4,5,6], healthcare [7], and transportation [8,9,10] by virtue of their efficient collaborative work mode. Collision avoidance control for human–robot collaboration is a safety guarantee for both humans and robots in human–robot cohesive environments [11,12]. In the process of robot executing a predetermined trajectory, the uncertainty of manual operation [13] as well as unpredictable objects on the predetermined trajectory [14] may cause the robot to collide, so improving the suppleness of the robot’s collision response and reducing the collision force have become urgent problems in robot safety control.

At present, numerous scholars have conducted in-depth research on unexpected collisions of robots and proposed different collision-response schemes [15,16]. Hu et al. designed a response method combining emergency braking and reverse motion by utilizing the force-sensing information of six-dimensional force sensors, and then presented different forms of response by comparing the angle between the end collision force and the end velocity [17]. Wang et al. proposed a zero-force control response strategy, through robot position control to torque control-switching so that the robot enters a zero-force state, to achieve the effect of conforming to a collision with an external forward force [18]. Sun et al. proposed a reflex control response strategy, which realizes the robot’s active disengagement from the collision position after collision by estimating the collision point position, the magnitude and direction of the collision force, and the joint velocity, thus avoiding secondary or continuous injuries to humans [19]. Nguyen et al. proposed a response method to compensate the current drift during human–robot collision, which utilizes a neural network controller to change the robot joint speed to compensate the current drift, thus reducing the interaction force generated by the collision [20]. Zhang et al. proposed a position-based overdamped collision control strategy to slow down spacecraft by applying a continuous control torque to slow down the rotational speed of the spacecraft, which reduces the collision impact when grasping and solves the problem that space robots have difficulty in capturing high-speed rotating spacecraft [21,22]. Dang et al. proposed a collision-response strategy for trial forward travel, which enabled the robot to avoid the problem of mission interruption by switching between three modes of high stiffness, low stiffness, and torque control [23]. Loris et al. proposed the application of a conductive controller to the robot collision response to realize the end-effector’s compliance with the collision force applied to it [24]. Zhou et al. utilized a variable stiffness conductivity control for collision response, updating the conductivity parameters based on the magnitude and direction of the collision external force to form an avoidance trajectory, which improves the flexibility of the collision response while guaranteeing the continuity of the task [25,26]. Kim et al. proposed an on-line computation of the inertial parameter of the response in the conductivity control to enable the robot joints to produce an avoidance trajectory without exceeding the maximum permissible torque [27].

As demonstrated in the aforementioned research, there are four primary forms of collision response implemented by robots, namely emergency braking, reflex control, control mode switching, and admittance control, yet all these existing response forms exhibit distinct limitations and struggle to balance safety, compliance, and task continuity [28]. Specifically, emergency braking refers to the immediate termination of the robot’s motion commands upon collision; boasting a fast response speed and simple logic, it is suitable for high-risk scenarios, but it fails to mitigate the impact force at the moment of collision, making it prone to rigid impacts. Reflex control achieves trajectory evasion based on collision force feedback, which can effectively reduce collision magnitude. It requires restarting the pre-planned trajectory after the collision subsides, inevitably causing task interruptions and thus struggling to adapt to the requirements of continuous operation. The control mode-switching method aims to address the issue of task interruption through seamless transitions between different operational modes, while the core bottleneck of such strategies lies in the transition smoothness, as impact is likely to occur during the mode-switching process. Admittance control stands out as a promising solution in human–robot collaboration scenarios, but current methods rely solely on adjusting a single parameter for collision response, which is often insufficient to achieve the desired compliance and flexibility required for safe human–robot interaction.

To address this issue, this paper proposes a collision-response algorithm based on adaptive admittance control, building upon the foundation of admittance control, with the aim of enhancing the compliance of robot collision response and resolving the problem of task interruption. By dynamically adjusting the stiffness and damping parameters of the collaborative robot, this method transforms “hard collisions” into “soft collisions,” effectively buffering instantaneous impact forces while ensuring the stability of the robot’s posture, thereby overcoming the limitation of traditional admittance control, which struggles to balance compliance and stability. Furthermore, the algorithm does not require the scheduled operation task to be interrupted during the collision-response process; instead, it generates a smooth obstacle-avoidance trajectory by adjusting admittance parameters in real-time and automatically reconnects to the original trajectory after the collision subsides. Simulation and physical platform experiments demonstrate that, compared with traditional control strategies, the proposed algorithm exhibits significant advantages in collision force suppression, trajectory smoothness, and task continuity, providing a more reliable safety control scheme for complex human–robot collaboration scenarios.

The research content of this paper is as follows: First, an admittance collision-response model is established, and the admittance parameters of the one-dimensional admittance model are simulated and analyzed to obtain the influence of each admittance parameter on system characteristics. Second, an adaptive admittance control collision-response algorithm is proposed and a simulation model is established, and this is compared with other admittance control response algorithms to verify the superiority of the proposed collision-response algorithm. Third, a collision-response platform is built to verify the effectiveness of the algorithm. Finally, a summary is made, the deficiencies in the research process are noted, and the future optimization directions are proposed.

## 2. Materials and Methods

### 2.1. Modeling of the Conductor and Analysis of the Conductor Parameters

**Figure 1:**
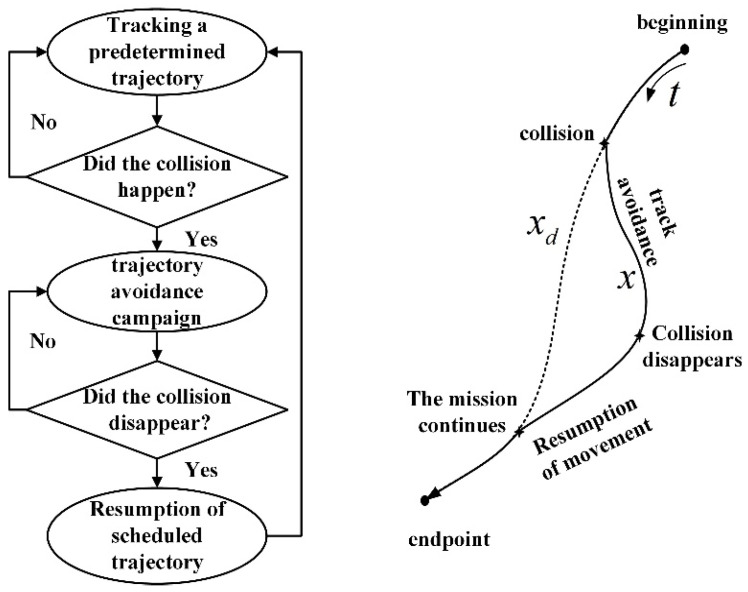
Schematic of robot end-effector collision response.

In the human–robot collaboration scenario, there are two main cases: robot end-effector collision and robot body collision. The following research will take the robot end collision as the entry point to build the conduction collision model. First of all, the end-effector collision-response process is defined, as shown in Figure 1, under normal circumstances: if the robot end does not sense the collision, it will follow the predetermined trajectory to carry out the human–robot collaboration task. Once the collision occurs, the robot system will quickly capture the collision force information as the basis for trajectory avoidance, thus reducing collision damage and achieving smooth control effects. When the collision is over, the robot then enters the recovery movement to ensure the consistency of the task, effectively preventing the task from being interrupted and guaranteeing the smooth operation of the whole collaboration process.

**Figure 2:**
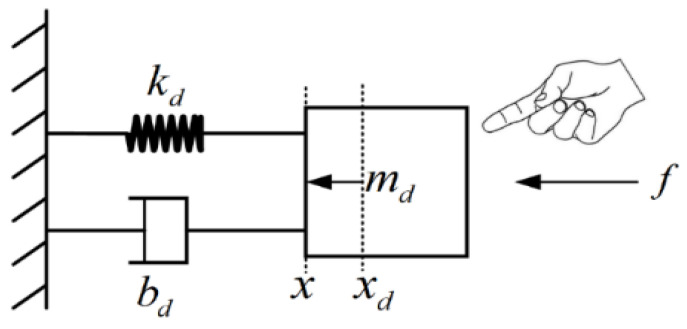
Second-order mass-damped–spring collision models.

Based on the admittance control theory, the robot is regarded as a second-order mass-damping–spring system, as shown in Figure 2, the second-order admittance collision model is established, and the admittance parameters are simulated and analyzed.

The second-order admittance collision model is represented as follows:

(1) $$m_{d}\left(\ddot{x}-{\ddot{x}}_{d}\right)+b_{d}\left(\dot{x}-{\dot{x}}_{d}\right)+k_{d}\left(x-x_{d}\right)=f$$

where denotes the actual trajectory displacement; $x_{d}$ denotes the target trajectory displacement; f denotes the collision external force information; $m_{d}$, $b_{d}$, and $k_{d}$ denote the virtual inertia parameter, virtual damping parameter, and virtual stiffness parameter, respectively.

From the above admittance model, when there is no collision external force on the second-order system, f = 0, x = $x_{d}$, the system moves along the target trajectory $x_{d}$; if a collision occurs, with the collision external force f ≠ 0, x ≠ $x_{d}$, the system generates an avoidance motion and forms an avoidance trajectory; at the end of the collision, with the collision external force f = 0, the second-order system is guided by the admittance control so that x gradually approaches $x_{d}$; when x = $x_{d}$, the system recovers the target trajectory. This leads to the conclusion that this admittance collision model conforms to the initially defined collision-response process.

Let $x_{e}$ = x − $x_{d}$, and perform the Laplace transform of Equation (1) to obtain its transfer function:

(2) $$h\left(s\right)=\frac{x_{e}\left(s\right)}{f\left(s\right)}=\frac{1}{m_{d}s^{2}+b_{d}s+k_{d}}\frac{n!}{r!\left(n-r\right)!}$$

**Figure 3:**
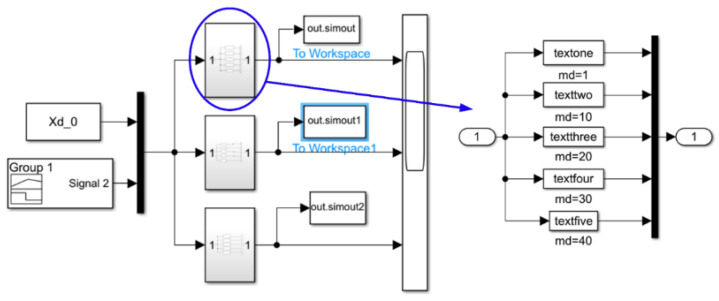
Simulation system for admittance parameters.

In the research on robot control systems, particularly in the simulation of admittance control and collision response, selecting an appropriate simulation platform is crucial [29]. Simulink version R2022a (9.12.0.1855793) can provide a unified and efficient platform for the co-simulation of the physical system and control algorithm in this study. Its seamless integration with MATLAB offers powerful data analysis capabilities [30,31]. Therefore, Simulink was adopted as the simulation environment. We performed the simulation using MATLAB version R2022a (9.12.0.1855793). The Simulink simulation system built using MATLAB is shown in Figure 3.

Since the admittance model of the collaborative robot is decoupled in the joint space as well as in the Cartesian space in each direction, a one-dimensional admittance model was used as an example, so that the collision force f = 10 N and the mass parameter $m_{d}$, the damping parameter $b_{d}$, and the stiffness parameter $k_{d}$ were simulated and analyzed according to the idea of controlling variables in the one-dimensional admittance model.

**Figure 4:**
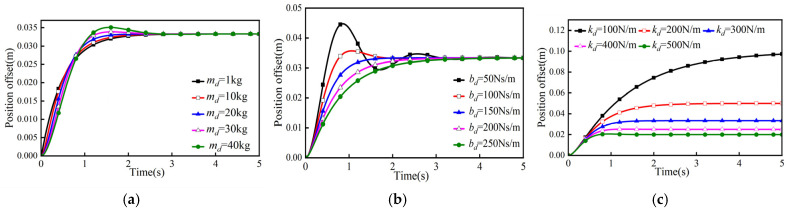
Parameter simulation results. (**a**) Mass parameter simulation results. (**b**) Damping parameter simulation results. (**c**) Stiffness parameter simulation results.

In simulation experiment 1, $b_{d}$ = 150 Ns/m, $k_{d}$ = 300 N/m, and mass parameters $m_{d}$ were 1 kg, 10 kg, 20 kg, 30 kg, and 40 kg; the simulation results are shown in Figure 4a.

In simulation experiment 2, $m_{d}$ = 20 kg, $k_{d}$ = 300 N/m, and damping parameters $b_{d}$ were 50 Ns/m, 100 Ns/m, 150 Ns/m, 200 Ns/m, and 250 Ns/m; the simulation results are shown in Figure 4b.

In simulation experiment 3, $m_{d}$ = 20 kg, $b_{d}$ = 300 N/m, and stiffness parameters $k_{d}$ were 100 Ns/m, 200 Ns/m, 300 Ns/m, 400 Ns/m, and 500 Ns/m; the simulation results are shown in Figure 4c.

According to the simulation results, when $b_{d}$ and $k_{d}$ are constant, the larger the value of $m_{d}$ in the one-dimensional admittance model, the stronger the inertia property of the system, and if the value of $m_{d}$ is too large, the phenomena of overshooting and oscillation will be produced; when $m_{d}$ and $k_{d}$ are constant, the smaller the value of $b_{d}$, the larger the output speed of the system, but if the value of $b_{d}$ is too small, the phenomena of overshooting and oscillation also occur; when $m_{d}$ and $b_{d}$ are constant, the $k_{d}$ in the system is negatively correlated with displacement deviation, and the larger the displacement deviation, the smaller the displacement deviation. When $m_{d}$ and $b_{d}$ are constant, $k_{d}$ and displacement deviation in the system are negatively correlated, and the larger $k_{d}$ is, the smaller the displacement deviation.

### 2.2. Adaptive Admittance Control of Collision-Response Strategies

The essence of adaptive admittance controller design is to rationally adjust the admittance parameters according to the robot’s task requirements to achieve the desired effect. Adaptive admittance control in collision response should ensure that it exhibits both proper suppleness and flexibility in the event of a collision at the end of the robot, as well as ensuring the accuracy of robot trajectory-tracking in the absence of a collision. The above requirements were analyzed using admittance parameter analysis in the admittance controller and through looking at adaptive adjustment.

From the analysis of admittance parameters, it can be seen that in the case of a certain collision force and other admittance parameters, the size of the value of $b_{d}$ will affect the response speed of the robot’s end, so the idea of adjusting $b_{d}$ is as follows.

When a collision occurs, the collision response manifests as acceleration in the direction of the collision force, resulting in an avoidance trajectory. In this case, a large damping parameter is clearly inappropriate, as this limits the acceleration and velocity of the robot. The damping parameter in this direction should be reduced at this point to ensure the flexibility of the robot end. Therefore, the damping adjustment expression based on the end crash response is defined as follows:

(3) $$b_{mi}=b_{md}-\alpha \left(f_{i}\right)$$

where

(4) $$\alpha \left(f_{i}\right)=\max\{\tilde{\alpha }\left(f_{i}\right),\;\beta \}$$

(5) $$\tilde{\alpha }\left(f_{i}\right)=\left\{\begin{array}{c} \;0\;\;\;f_{i}\leq \delta_{m}\\ a_{1}\left|f_{i}\right|\;\;f_{i}>\delta_{m} \end{array}\right.$$

where $b_{mi}$ denotes the robot end variable damping parameter; $b_{md}$ denotes the robot end virtual damping parameter; $\tilde{\alpha }\left(f_{i}\right)$ denotes the damping change term; β denotes the minimum value of the damping variation term $\tilde{\alpha }\left(f_{i}\right)$; $\delta_{m}$ denotes the collision force/moment threshold at the end of the robot; $f_{i}$ is the force component of $f_{e}$ on the x-, y-, and z-axes (Cartesian space); $a_{1}$ denotes the damping adjustment weighting factor.

In the case of a certain collision force and other guiding parameters, the size of $k_{d}$ directly affects the displacement deviation of the robot, which in turn affects the suppleness of the collision response, so the idea of adjusting $k_{d}$ is as follows.

When a collision does not occur, the robot moves according to a predetermined trajectory so that the robot has a large stiffness parameter, which reduces oscillations caused by sensor noise and improves trajectory-tracking accuracy; When a collision occurs, the stiffness parameter $k_{d}$ is reduced as the collision force $f_{i}$ increases. With a smaller stiffness parameter, the robot end produces a larger, more supple avoidance displacement in the direction of the collision force. When the collision ends, the collision force $f_{i}$ disappears and the robot returns to the state of the large stiffness parameter, where the robot is constrained by the reference trajectory to increase, forcing x to gradually approach $x_{d}$, thus restoring the intended trajectory.

First, the stiffness adjustment function based on the robot end collision response is defined:

(6) $$\chi =\tanh({f_{i}}^{2})$$

The value of χ is restricted to the interval [0, 1) by the “tanh” function, and the function is continuous without mutation.

When there is no force acting in any direction x is 0, and when there is a collision force acting x approaches 1, based on which the stiffness parameter $k_{d}$ of the conductivity control system is redefined.

(7) $$k_{mi}=\left(1-a_{2}\chi \right)k_{md}$$

where $k_{mi}$ denotes the end variable stiffness parameter; $a_{2}$ denotes the weighting factor. Up to this point, the one-dimensional admittance model is rewritten according to the redefined variable damping parameter $b_{mi}$ and variable stiffness parameter $k_{mi}$ to obtain an adaptive admittance control model of the robot end-crash response:

(8) $$m_{md}{\ddot{x}}_{e}+b_{mi}{\dot{x}}_{e}+k_{mi}x_{e}=f_{i}$$

where $x_{e}$ denotes the displacement deviation.

**Figure 5:**
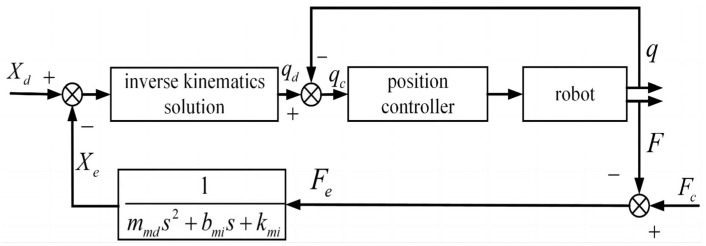
Block diagram of adaptive admittance control for robot end collision response.

From the above theoretical derivation, an adaptive admittance control system for robot end collision response can be derived, as shown in Figure 5.

Similarly, the above analysis of the admittance parameters of the robot end–end collision response also applies to the robot joint collision response. By replacing the collision force information $f_{i}$ of the robot’s end response with the collision moments $\tau_{i}^{p}$ at each joint, an expression for the damping adjustment of the collision response of the robot’s joints can be obtained:

(9) $$b_{ji}=b_{jd}-\alpha \left(\tau_{i}^{p}\right)$$

where $b_{ji}$ denotes the joint variable damping parameter and $b_{jd}$ denotes the joint virtual damping parameter.

The stiffness adjustment expression for the collision response of the robot joints is given by

(10) $$k_{ji}=\left(1-a_{2}\chi \right)k_{jd}$$

where $k_{ji}$ denotes the joint variable stiffness parameter and $k_{jd}$ denotes the joint virtual stiffness parameter.

In this way, the adaptive admittance control model for the collision response of the robot joints is obtained as

(11) $$m_{jd}{\ddot{q}}_{ie}+b_{ji}{\dot{q}}_{ie}+k_{ji}q_{ie}=\tau_{i}^{p}$$

where $q_{ie}$ denotes the angular difference in the joint i.

**Figure 6:**
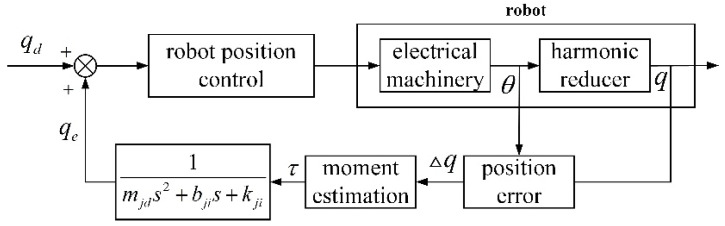
Block diagram of adaptive admittance control for the collision response of robot joints.

From the above theoretical derivation, an adaptive admittance control system for the collision response of the robot joints can be obtained, as shown in Figure 6.

As for the robot end, for example, the transfer function of the end adaptive admittance control model is

(12) $$h\left(s\right)=\frac{e_{i}\left(s\right)}{f_{i}\left(s\right)}=\frac{\frac{1}{m_{md}}}{s^{2}+\frac{b_{mi}}{m_{md}}s+\frac{k_{mi}}{m_{md}}}$$

The undamped natural frequencies and damping ratios of the above adaptive admittance system are shown below:

(13) $$\omega_{n}=\sqrt{\frac{k_{mi}}{m_{d}}}$$

(14) $$\xi =\frac{b_{mi}}{2\sqrt{m_{md}k_{mi}}}$$

In the above adaptive admittance system, the values of $m_{md}$, $b_{mi}$, and $k_{mi}$ need to satisfy 0 < ξ < 1 to ensure the stability of the system.

The obstacle-avoidance trajectory generation strategy based on adaptive admittance control is presented in Algorithm 1. This strategy calculates admittance parameters from collected collision forces, solves for second-order admittance model offsets, and synthesizes real-time avoidance trajectories. Additionally, an obstacle detection strategy based on force feedback is detailed in Algorithm 2. This utilizes a dual mechanism of ‘force signal monitoring’ and ‘active micro-motion backtracking detection’ to determine the continued presence of an obstacle, thereby effectively mitigating the risk of secondary collisions.
**Algorithm 1.** Algorithmic procedure for obstacle-avoidance trajectory generation based on adaptive admittance control**Input:** Desired trajectory $X_{d}$; Real-time feedback force $f_{i}$;
**Output:** Corrected real-time avoidance trajectory $X_{r}$;1**while** robot task is in execution **do**2
Acquire current collision force components $f_{i}$;3
Calculate variable parameters: Solve for $b_{mi}$ and $k_{mi}$ in real-time (Equations (3)–(7));4
Solve for deviation: Calculate displacement deviation $x_{e}$ using the admittance equation (refer to Equation (8));5
Trajectory synthesis: Compute $X_{r}=X_{d}+x_{e}$ as control output;6
Stability monitoring: Ensure current parameters satisfy 0<ξ<1 (Equation (14));7
Execution update: Transmit $X_{r}$ to the low-level controller;8**end while**
**Algorithm 2.** Strategy for obstacle presence determination and task recovery based on force feedback**Input:** Real-time force feedback $f_{i}$; Collision threshold $\delta_{m}$; Original parameters $\left\{b_{md},k_{md}\right\}$; **Output:** Restored system parameters and trajectory;1**if** sustained contact force $\left|f_{i}\right|>\delta_{m}$ **then**
2
Maintain state: Output current avoidance parameters $b_{mi}$, $k_{mi}$ and maintain avoidance pose;3**else** (Obstacle removal determined: $\left|f_{i}\right|\leq \delta_{m}$ and stable)4
Smooth recovery: Guide variable parameters back to initial values $\left\{b_{md},k_{md}\right\}$ (Equations (6) and (7));5
Trajectory reset: Output displacement deviation $e$ approaching 0 to return to desired trajectory $x_{e}\to 0$;6**end if**

### 2.3. Stability and Convergence Analysis

For the second-order adaptive admittance control system:

$$m_{md}{\ddot{x}}_{e}+b_{mi}{\dot{x}}_{e}+k_{mi}x_{e}=f_{i}$$

When the collision ends ($f_{i}=0$), the system simplifies to

$$m_{md}{\ddot{x}}_{e}+b_{mi}{\dot{x}}_{e}+k_{mi}x_{e}=0$$

This is a second-order system with time-varying parameters because $b_{mi}(t)$ and  (t) vary over time (adaptive adjustment).

Constructing a Lyapunov function, define the state variables: Define the state vector $x={\left[\begin{array}{cc} x_{e} & {\dot{x}}_{e} \end{array}\right]}^{T}$. Then, the system can be expressed in state-space form:

(15) $$\dot{x}=\left[\begin{array}{cc} 0 & 1\\ -\frac{k_{mi}(t)}{m_{md}} & -\frac{b_{mi}(t)}{m_{md}} \end{array}\right]x+\left[\begin{array}{c} 0\\ \frac{1}{m_{md}} \end{array}\right]f_{i}$$

**Theorem** **1** (Closed-loop System Stability)**.** *For the adaptive admittance control system described by Equation (8), if the adaptive parameters satisfy the following conditions:*

*There exist positive constants*$\;k_{min}$*,* $k_{max}$*,* $b_{min}$*,* $b_{max}$ *such that for any* t≥0*,* $k_{min}\leq k_{mi}(t)\leq k_{max}$*,* $b_{min}\leq b_{mi}(t)\leq b_{max}$.

*The rate of change in the stiffness parameter is bounded, i.e., there exists a constant*  γ>0 *, such that* $|{\dot{k}}_{mi}(t)|\;\leq \gamma k_{mi}(t)$*, and* $\gamma <\frac{2b_{min}k_{min}}{m_{md}k_{max}}$.

*The external collision force is bounded:*$\;|f_{i}(t)|\leq F_{max}$.

*Then the system is uniformly ultimately bounded (UUB) stable, and there exist positive constants* α*, *β*,* κ* such that the state error satisfies* 

(16) $$∥x(t)∥\leq \beta ∥x(0)∥e^{-\alpha t}+\frac{\kappa }{\alpha }F_{max}$$

*Choose the Lyapunov function candidate:*

(17) $$\begin{array}{cccc} & V(x,t)=\frac{1}{2}k_{mi}(t)x_{e}^{2}+\frac{1}{2}m_{md}{\dot{x}}_{e}^{2}+\epsilon x_{e}{\dot{x}}_{e} & & \end{array}$$

*where* ϵ *is a positive constant satisfying* $0<\epsilon <\sqrt{k_{min}m_{md}}$ *. It is straightforward to verify that*  V(x,t) *is positive definite.*

*Computing the time derivative of* V *along the system trajectories:*

(18) $$\begin{array}{cc} \dot{V} & =\frac{1}{2}{\dot{k}}_{mi}(t)x_{e}^{2}+k_{mi}(t)x_{e}{\dot{x}}_{e}+m_{md}{\dot{x}}_{e}{\ddot{x}}_{e}+\epsilon {\dot{x}}_{e}^{2}+\epsilon x_{e}{\ddot{x}}_{e}\\ & =\frac{1}{2}{\dot{k}}_{mi}(t)x_{e}^{2}+k_{mi}(t)x_{e}{\dot{x}}_{e}+{\dot{x}}_{e}[-b_{mi}(t){\dot{x}}_{e}-k_{mi}(t)x_{e}+f_{i}]\\ & +\epsilon {\dot{x}}_{e}^{2}+\epsilon x_{e}[-\frac{k_{mi}(t)}{m_{md}}x_{e}-\frac{b_{mi}(t)}{m_{md}}{\dot{x}}_{e}+\frac{1}{m_{md}}f_{i}]\\ & =-b_{mi}(t){\dot{x}}_{e}^{2}+\frac{1}{2}{\dot{k}}_{mi}(t)x_{e}^{2}+\epsilon {\dot{x}}_{e}^{2}-\epsilon \frac{k_{mi}(t)}{m_{md}}x_{e}^{2}-\epsilon \frac{b_{mi}(t)}{m_{md}}x_{e}{\dot{x}}_{e}\\ & +(1+\frac{\epsilon x_{e}}{m_{md}{\dot{x}}_{e}}){\dot{x}}_{e}f_{i}(\mathrm{assuming}\;{\dot{x}}_{e}\neq 0) \end{array}$$

*Using Young’s inequality* $|x_{e}{\dot{x}}_{e}|\;\leq \frac{\delta }{2}x_{e}^{2}+\frac{1}{2\delta }{\dot{x}}_{e}^{2}$ *(where*  δ>0 *) and the assumed conditions, we obtain, after rearrangement:*

(19) $$\begin{array}{cccc} & \begin{array}{cc} \dot{V} & \leq -[b_{min}-\epsilon -\frac{\epsilon b_{max}}{2\delta m_{md}}]{\dot{x}}_{e}^{2}\\ & -[\epsilon \frac{k_{min}}{m_{md}}-\frac{\gamma }{2}k_{max}-\frac{\epsilon b_{max}\delta }{2m_{md}}]x_{e}^{2}+\eta |f_{i}| \end{array} & & \end{array}$$

*where* η>0 *is a constant. By choosing sufficiently small* ϵ *and*  δ *such that both bracketed terms are positive, denoted as* $\;\alpha_{1}$ *and* $\alpha_{2}$ *, and letting* $\;\alpha =min(\alpha_{1},\alpha_{2})$ *, we have*

(20) $$\begin{array}{cccc} & \dot{V}\leq -\alpha V+\eta |f_{i}| & & \end{array}$$

*Applying the comparison lemma yields*

(21) $$\begin{array}{cccc} & V(t)\leq V(0)e^{-\alpha t}+\frac{\eta }{\alpha }F_{max} & & \end{array}$$

*When the collision ends (* $f_{i}=0$ *), the system states converge exponentially to the origin, i.e.,*

(22) $$\underset{t\to \infty }{lim}x_{e}(t)=0,\underset{t\to \infty }{lim}{\dot{x}}_{e}(t)=0$$

*This property guarantees that the robot can precisely recover to the preplanned task trajectory.*

*In summary, under reasonable parameter design—specifically, ensuring that the minimum damping setting satisfies* $b_{md}-\beta \geq b_{min}>0$*, where*  β *is the maximum value of the damping variation term, and ensuring via the adjustment factor* $\;a_{2}$ *that* $\;|{\dot{k}}_{mi}(t)|$ *meets the theorem’s condition—the proposed adaptive admittance control algorithm can guarantee the stability of the collision-response process.*

## 3. Experimental Design and Results

### 3.1. General Overview of the Collision-Response Experimental Platform for Human–Machine Collaboration

**Figure 7:**
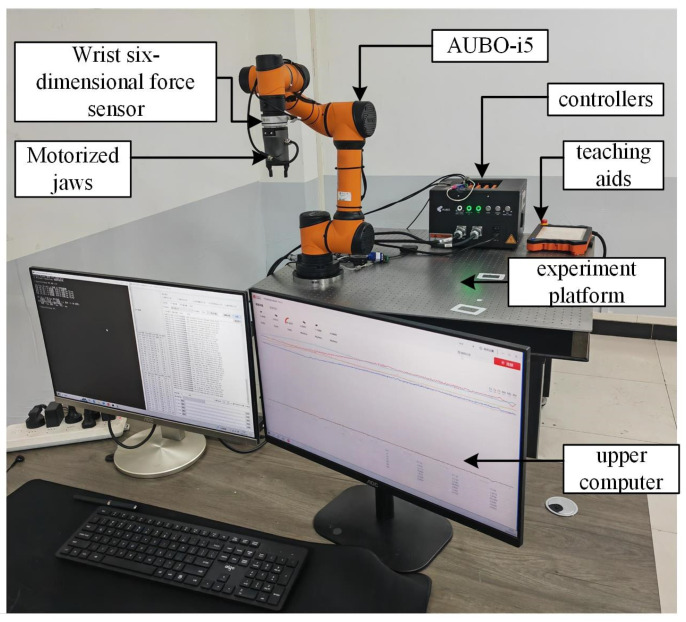
Collision-response experimental platform for human–machine collaboration.

In order to further validate the effectiveness of the collision detection method and collision-response strategy of the collaborative robot proposed in this paper, a human–robot collaborative collision-response platform was constructed, as shown in Figure 7, which consists of the AUBO-i5 collaborative robot, the base six-dimensional force sensor, the wrist six-dimensional force sensor, the end-effector (two-fingered gripper jaws), the upper computer, and the robot control cabinet with the demonstrator and other parts.

### 3.2. Response Architecture and Software–Hardware Integration Logic

**Figure 8:**
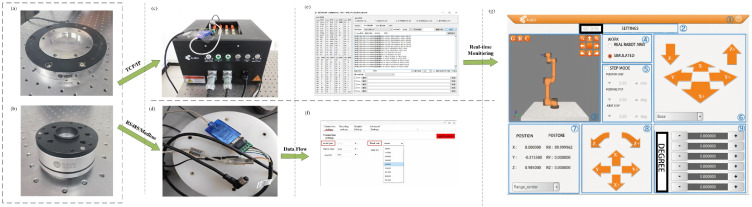
System integration architecture.

The system integration architecture for the proposed collision-response mechanism is illustrated in Figure 8. A multi-dimensional force feedback network was constructed by integrating data from KWR200X/KWR75D (Manufactured by Changzhou Kunwei Sensing Technology Co., Ltd., Changzhou, China) high-precision external six-axis force sensors with the robot’s internal joint encoders. A customized communication layer was developed to extract joint module data in real-time via Socket-TCP/IP (Port 8002), while external force sensor data were synchronized through a high-speed RS485 link to ensure the precise time-stamping of the algorithm inputs. The proposed adaptive admittance algorithm was embedded into the robot control loop via the Modbus RTU protocol. Rather than executing a simple ‘Emergency Stop’, the system achieves compliant avoidance by modifying joint target position registers in real-time. Upon the disappearance of the collision force, a script-driven RET function is utilized to facilitate automatic task trajectory recovery.

### 3.3. Triggering Thresholds and Response Latency

The built-in protection mechanism of the AUBO-i5 robot is primarily designed for destructive collisions under a 5 kg rated load, with a triggering threshold typically set between 5 and 10 N·m. In contrast, the algorithm presented in this study utilizes high-sensitivity feedback to set the collision force triggering threshold at 0.2 N (corresponding to a torque threshold of 0.2 N·m). This difference in magnitude ensures that the algorithm responds prior to the built-in mechanisms. Furthermore, while the system-level delay of the robot’s built-in emergency stop is typically over 100 ms, the adaptive response cycle of the proposed algorithm is only 10–50 ms. This allows for avoidance maneuvers via microsecond-level trajectory corrections before the built-in mechanism detects a high-magnitude impact.

### 3.4. Simulation of Crash Response Algorithm for Adaptive Admittance Control

#### 3.4.1. Simulation of End–End Collision Response of Collaborative Robots

**Figure 9:**
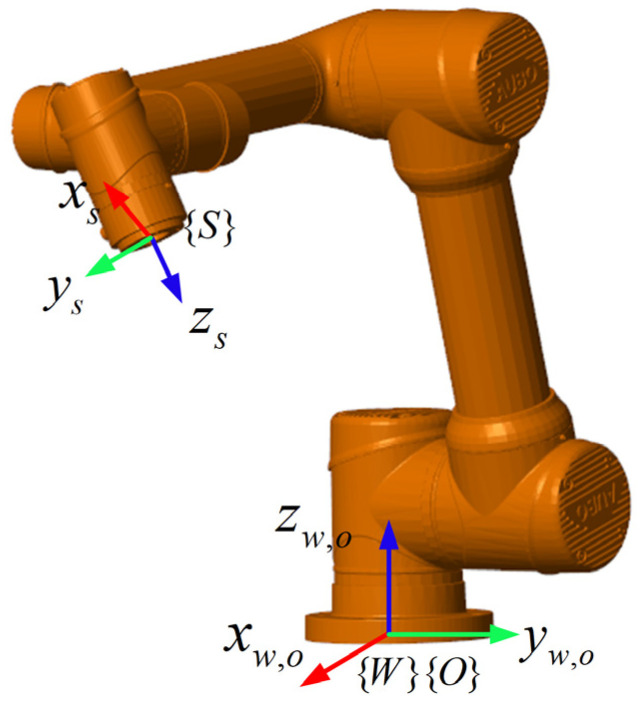
AUBO-i5 Simscape simulation model.

First, a Simscape 3D physical simulation model of the AUBO-i5 robot is built, as shown in Figure 9, and the algorithm is verified in MATLAB, where {W} and {O} are the world coordinate system and base coordinate system in the simulation environment, respectively, and {S} is the end-effector coordinate system. The collision phenomenon during human–robot collaboration is simulated by applying a collision force in the end-effector coordinate system {S}.

Define the intended task trajectory of the collaborative robot in the world coordinate system {W} as a circle trajectory in space centered at point a with normal vector $\overset{⃑}{n}=\left(-1,-1,-0.3\right)$ and radius r = 200. The simulation time is set to 20 s, the sampling time is 0.002 s, the distance unit is in millimeters, and the joint angle of the collaborative robot is expressed in radians.

In the following simulation verification, the adaptive admittance controller is simulated using the end collision force information of the collaborative robot to verify the suppleness and flexibility of the adaptive admittance control proposed in this paper in the collision response of the robot’s end-effector, and to compare it with the variable stiffness admittance control as well as the fixed-parameter admittance control. Because the avoidance trajectory generated by the collision response of the robot end is mainly formed by the change in the robot end position in the human–robot collaboration task, and the robot end attitude has less influence on the avoidance trajectory, this paper is aimed at the simulation validation of the admittance model of the robot end in the axis direction.

**Figure 10:**
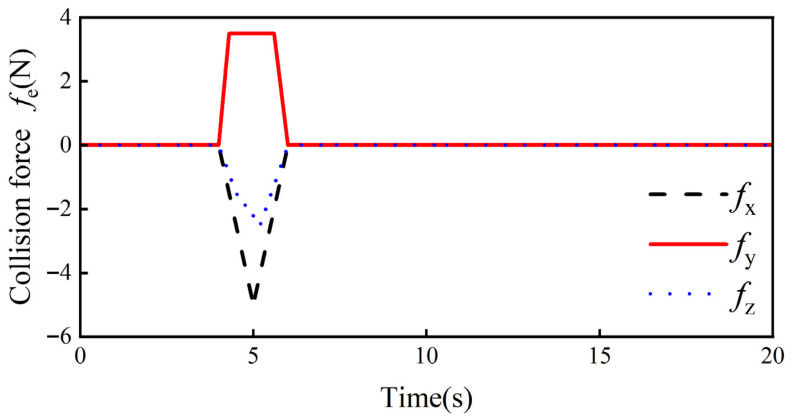
Robot end collision force $f_{e}$.

At first, the collision force when a collision occurs at the end of the robot is defined. Since the human–robot collaborative collision avoidance control system proposed in this paper detects collisions through force feedback, the collision force is assumed to be a constant value, and the suppleness and flexibility of the robot’s collision response are determined by comparing the avoidance trajectories generated by different control algorithms. Without considering the effect of the robot’s end pose on the avoidance trajectory, the torque applied to the x-, y-, and z-axes is set to 0, and it is hypothesized that the robot is subjected to a collision force $f_{e}$ = [$f_{x}$, $f_{y}$, $f_{z}$] T as shown in Figure 10 at the 4 s of the simulation, where the magnitude and the direction of $f_{x}f_{y}$, and fz are given by Equations (15), (16) and (17), respectively:

(23) $$f_{x}=\left\{\begin{array}{cc} -5t+20 & 4\leq t<5\\ 5t-30 & 5\leq t\leq 6\\ 0 & t<4\;or\;t>6 \end{array}\right.$$

(24) $$f_{y}=\left\{\begin{array}{cc} \frac{35}{3}t-\frac{140}{3} & 4\leq t<4.3\\ 3.5 & 4.3\leq t<5.6\\ -8.75t+52.5 & 5.6\leq t\leq 6\\ 0 & t<4\;or\;t>6 \end{array}\right.$$

(25) $$f_{z}=\left\{\begin{array}{cc} -3t+12 & 4\leq t<4.5\\ -\frac{10}{7}t+\frac{69}{14} & 4.5\leq t<5.2\\ 3.125t-18.75 & 5.2\leq t\leq 6\\ 0 & t<\;or\;t>6 \end{array}\right.$$

**Figure 11:**
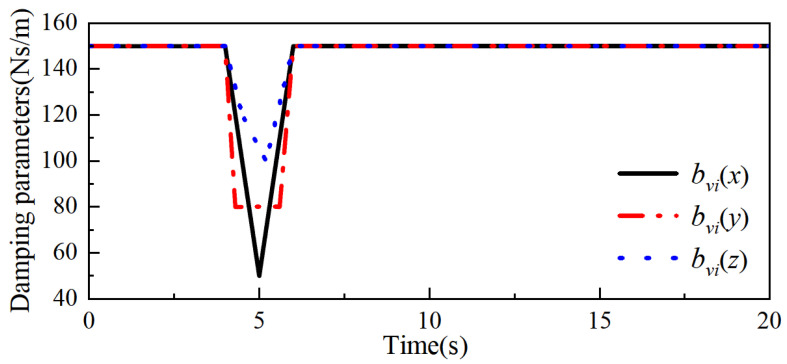
$b_{vi}$ variation curve.

**Figure 12:**
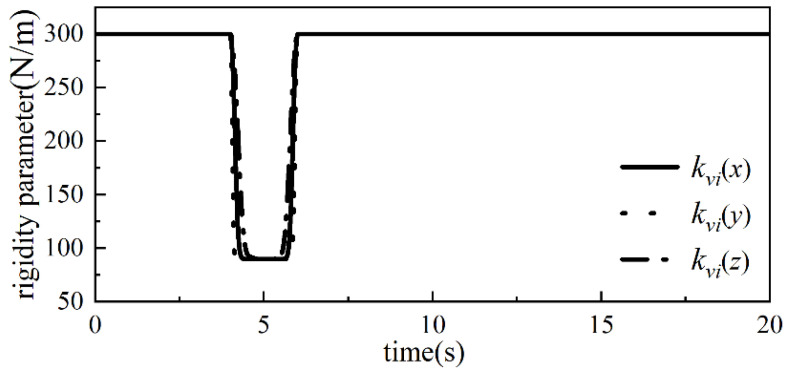
$k_{vi}$ variation curve.

Secondly, the ideal admittance parameters $m_{md}$ = 20 kg, $b_{md}$ = 150 Ns/m, and $k_{md}$ = 300 N/m in the x-, y-, and z-axes of the robot end are determined through several simulation experiments, and the weighting factors $a_{1}$, $a_{2}$, and β in the adaptive admittance strategy are $a_{1}$ = 20, $a_{2}$ = 0.7, and β = 30 Ns/m, with the collision force threshold value δ = 0.2 N. Combining the above control parameters and the collision force information can obtain the $b_{mi}$ change curve and $k_{mi}$ change curve in the adaptive admittance strategy, which are shown in Figure 11 and Figure 12, respectively.

**Figure 13:**
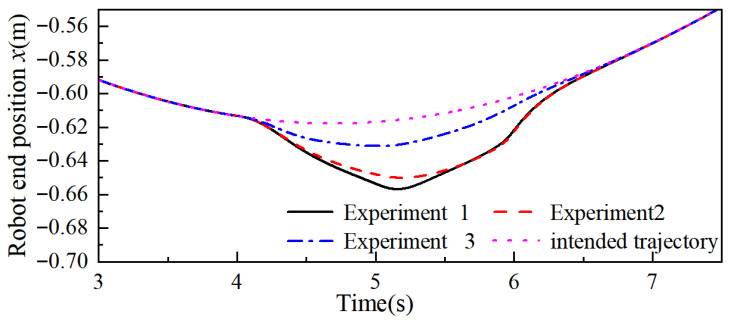
Robot end x-axis trajectory.

**Figure 14:**
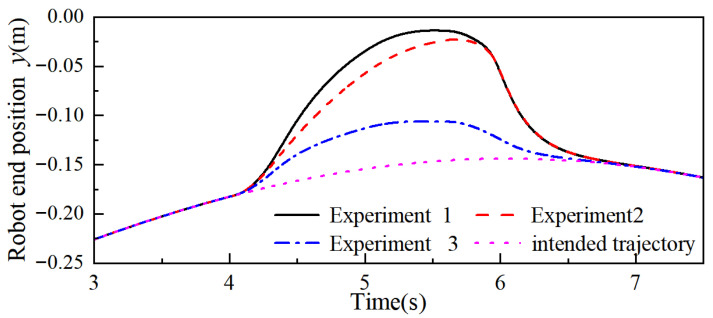
Robot end y-axis trajectory.

**Figure 15:**
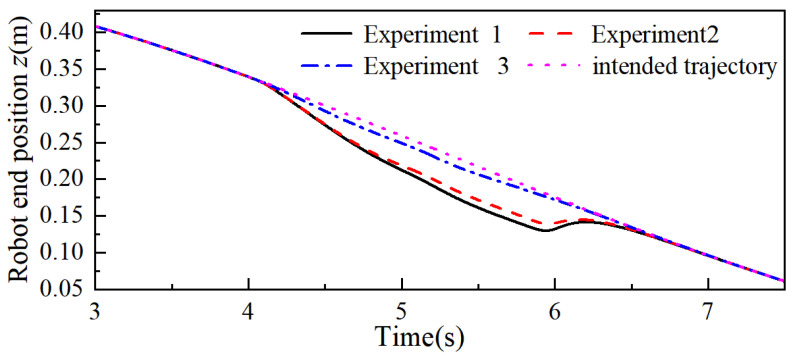
Robot end z-axis trajectory.

Then, the adaptive admittance control proposed in this paper is set as Experiment 1, the variable stiffness admittance control is set as Experiment 2, and the fixed-parameter admittance control is set as Experiment 3, respectively, to conduct controlled simulation experiments. The simulation results are shown in Figure 13, Figure 14 and Figure 15, where only the end trajectory variations within 3–7.5 s are retained for clarity of display. During 3–4 s, $f_{e}$ = 0, the robot moves along the predefined task trajectory; at 4 s the robot end is subjected to a collision force $f_{e}$, and the robot starts the collision response to generate the avoidance trajectory; at 6 s the collision force disappears, the collision ends, and the robot gradually recovers the predefined task trajectory under the guidance of the admittance control.

From Figure 13 and Figure 15, it can be seen that the maximum positional offsets of the robot end in the x-, y-, and z-axes in Experiment 1 were 41.30 mm, 133.35 mm, and 36.72 mm; in Experiment 2, this data was 35.16 mm, 122.47 mm, and 32.47 mm; and in Experiment 3, this data was 14.87 mm, 43.09 mm, and 11.79 mm. By comparing the end position offset, it can be seen that when the robot carries out the collision response, the two control methods of changing the admittance parameters are more effective than the fixed-parameter admittance control, which produces a larger avoidance trajectory, and this illustrates the practical significance of the reasonable change in the admittance parameters in the process of collision response.

The response time when the robot end produces the maximum positional offset in the x-, y-, and z-axes in Experiment 1 is 5.186 s, 5.406 s, and 5.460 s, respectively; in Experiment 2, this data is 5.298 s, 5.652 s, and 5.592 s, and through combination with the robot end positional offset, it can be concluded that the maximum positional offset in the x-, y-, and z-axes for the end in Experiment 1 compared with that in Experiment 2 increased by 17.46%, 8.89%, and 13.09%, respectively, and the response time decreased by 7.94%, 14.89%, and 8.21%, respectively. With the same collision force $f_{e}$, stiffness parameter $k_{vi}$, the adaptive admittance control proposed in this paper gives the robot better flexibility and suppleness, and varying the damping parameter makes the algorithm respond faster than the variable stiffness admittance control and produces larger avoidance trajectories. Similarly, when the avoidance trajectory required at the end of the robot is certain, the algorithm can reduce the impact force generated by the collision and provide safety for human–robot collaboration tasks.

#### 3.4.2. Simulation of Collision Response of Collaborative Robot Body

**Figure 16:**
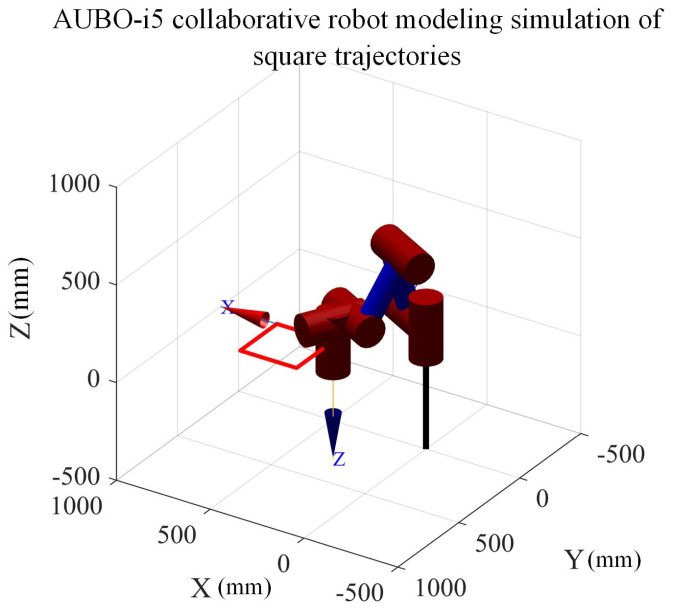
AUBO-i5 collaborative robot model square path simulation.

First, the robot task trajectory is defined as a square path in the plane in the world coordinate system w of the collaborative robot. Considering that subsequent experiments require the installation of a six-dimensional force sensor and end-effector at the end of the robot at the wrist, the trajectory plane is set to be 100 mm in the positive direction of the XW, O-axis, in the world coordinate system {W}. The starting coordinates of this square trajectory are (300, 300, 100) and the side length is 300 mm, as shown in Figure 16. The simulation time is set to 10 s, the sampling interval is 0.002 s, the distance unit is in millimeters, and the joint angle is expressed in radians.

The collaborative robot body collision-response simulation experiment adopts the same control experiment method as the previous end collision-response experiment. The experiments are divided into three groups: experiment 1 uses the adaptive admittance control response method proposed in this paper, experiment 2 uses the variable stiffness admittance control response method, and experiment 3 uses the fixed-parameter admittance control response method. Under the condition of the same collision force information, the avoidance trajectories generated by different control algorithms are compared to evaluate the suppleness and flexibility of the robot’s collision response. The difference between the ontology collision-response simulation experiment and the end collision-response experiment is that the input of the end collision response is the collision force from the direction of the x, y, and z coordinate axes, while the input of the ontology collision response is the collision moment from each joint.

**Figure 17:**
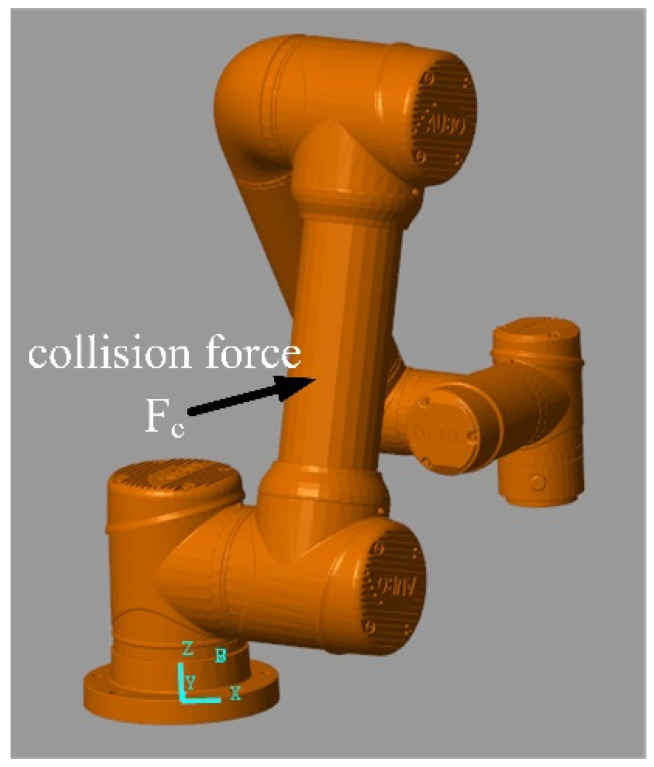
AUBO-i5 collaborative robot big arm collision diagram.

The simulation process takes the collision of the big arm joints of the AUBO-i5 collaborative robot as an example. When the big arm of the collaborative robot collides, not only joint 2 will generate collision moments, but joint 1 will also be affected by the collision force component and generate collision moments, as shown in Figure 17.

**Figure 18:**
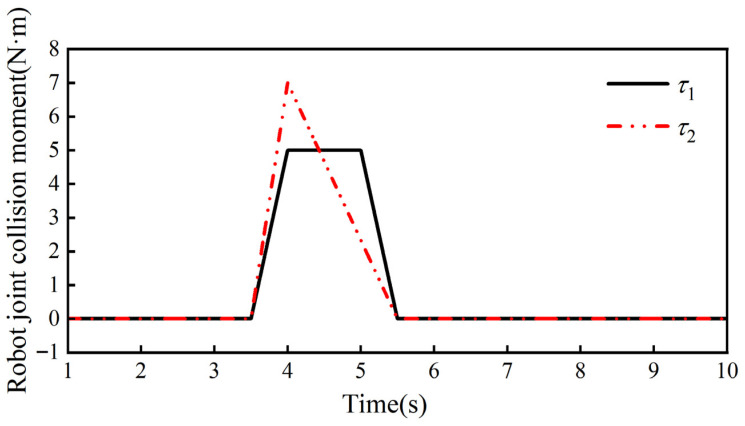
Collision simulation moments for collaborative robots.

Thus, it is assumed that the robot is subjected to a collision moment $\tau_{e}=\left[\begin{array}{cccccc} \tau_{1} & \tau_{2} & \tau_{3} & \tau_{4} & \tau_{5} & \tau_{6} \end{array}\right]$, as shown in Figure 18, at 3.5 s in the simulation; because the collision occurs with the big arm of the collaborative robot, so $\tau_{3}$, $\tau_{4}$, $\tau_{5}$, and $\tau_{6}$ are all 0, and the magnitude and the direction of $\tau_{1}$ and $\tau_{2}$ are given by Equations (18) and (19), respectively:

(26) $$\tau_{1}=\left\{\begin{array}{cc} 10t-35 & 3.5\leq t<4\\ 5 & 4\leq t<5\\ -10t+55 & 5\leq t\leq 5.5\\ 0 & t<3.5\;or\;t>5.5 \end{array}\right.$$

(27) $$\tau_{2}=\left\{\begin{array}{cc} 10t-35 & 3.5\leq t<4\\ 5 & 4\leq t<5\\ -10t+55 & 5\leq t\leq 5.5\\ 0 & t<3.5\;or\;t>5.5 \end{array}\right.$$

**Figure 19:**
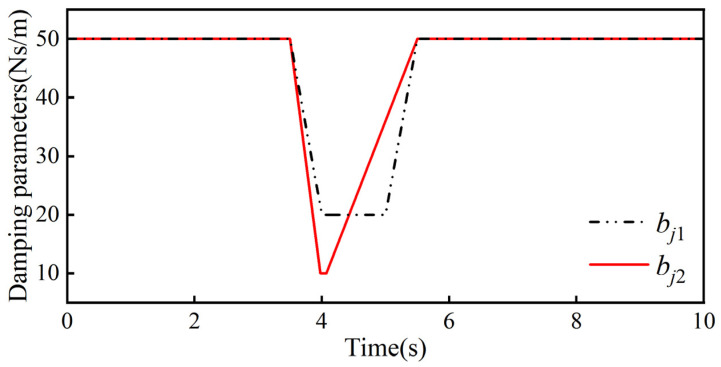
$b_{ji}$ change curve.

**Figure 20:**
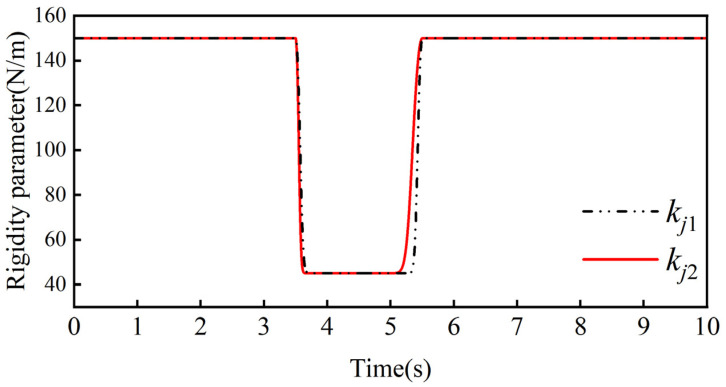
$k_{ji}$ change curve.

Through several simulation experiments, the ideal admittance parameters in the robot joint admittance were determined to be $m_{jd}$ = 30 kg, $b_{jd}$ = 40 Ns/m, and $k_{jd}$ = 150 N/m, the weighting factors in the adaptive admittance strategy are $a_{1}$ = 6, $a_{2}$ = 0.7, and β = 10 Ns/m, and the threshold value of the collision force is δ = 0.2 N. Combining the above control parameters with the collision moments, we can obtain the $b_{ji}$ variation curve and $k_{ji}$ variation curve in the adaptive admittance strategy, which are shown in Figure 19 and Figure 20, respectively.

**Figure 21:**
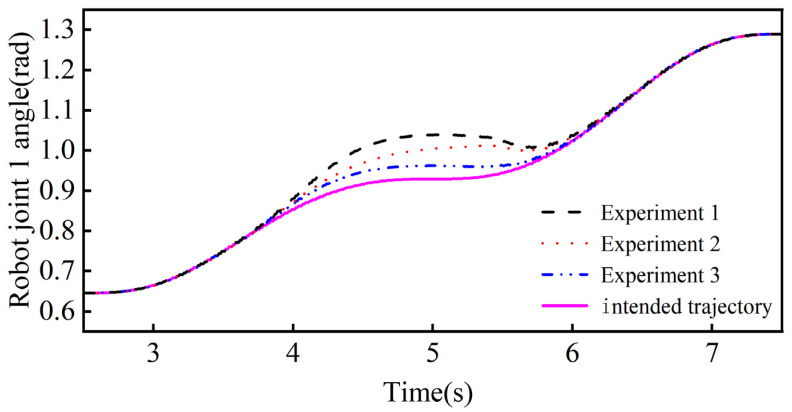
Joint 1 angle in the simulated control test.

**Figure 22:**
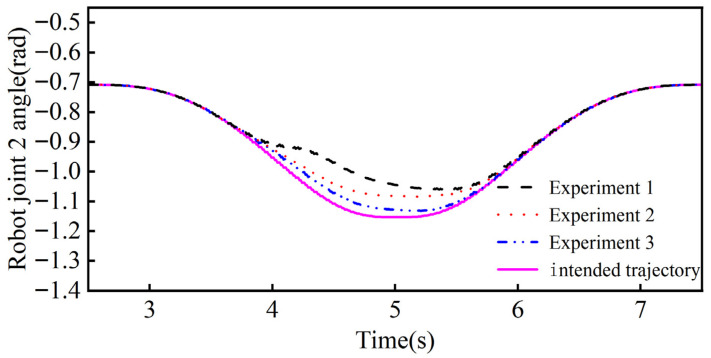
Joint 2 angle in the simulated control test.

The results of the collision-response simulation experiments of the collaborative robot body are shown in Figure 21 and Figure 22. From Figure 21, it can be seen that the maximum angular offsets of joint 1 in the three groups of controlled simulation experiments are 0.1102 rad, 0.0790 rad, and 0.0329 rad, and the response times for generating the maximum angular offsets are 4.964 s, 5.072 s, and 5.218 s. The response times for generating the maximum angular offsets are 4.964 s, 5.072 s, and 5.218 s. From Figure 22, it can be seen that the maximum angular offsets of joint 2 in the three groups of controlled simulation experiments are 0.1387 rad, 0.0828 rad, and 0.0361 rad, respectively, and the response times for generating the maximum angular offsets are 4.524 s, 4.658 s, and 4.844 s, respectively.

The above data shows that in the maximum angular offset of joint 1 the adaptive admittance control response method proposed in this paper is improved by 39.49% and 234.95% and reduces by 7.16% and 14.78% in response time compared to the variable stiffness admittance control response method and fixed-parameter admittance control response method, respectively. The data from Joint 2 likewise show that adaptive admittance control has significant advantages in increasing the avoidance angle offset and reducing the response time. The simulation results verify the applicability of the adaptive admittance control method in robot body collision response and demonstrate its superior performance.

### 3.5. Collaborative Robot Collision-Response Experiment

#### 3.5.1. Collaborative Robot End Collision-Response Experiment

**Figure 23:**
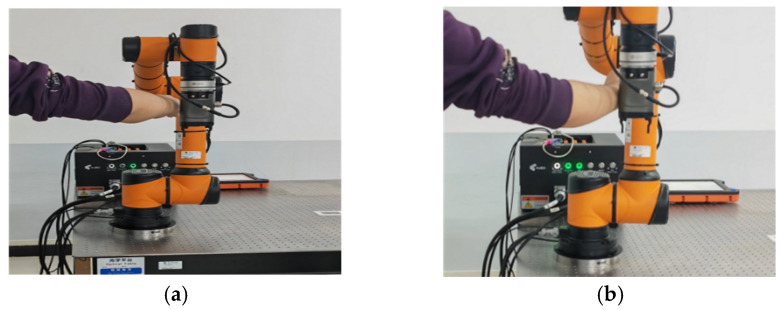

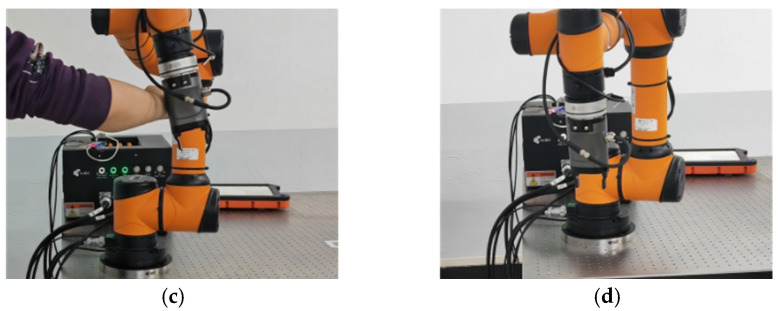
Robot end collision-response process. (**a**) Pre-collision. (**b**) Collision. (**c**) Collision response. (**d**) Resumption of mission trajectory.

The experiment utilizes the AUBO-i5 robot SDK development environment to define the robot end predetermined task trajectory and the human–robot collision response algorithm, and utilizes the KWSenserLinker v1.0 software to obtain the collision force information detected by the six-dimensional force sensor, KWR75D, and to gravity-compensate the robot end at the beginning of the experiment. The other experimental parameters during the experiment were the same as those in the simulation, and the volunteers used their hands to apply an external force on the end to simulate the robot end-effector collision experiment. The AUBO-i5 robot collision-response process is shown in Figure 23.

**Figure 24:**
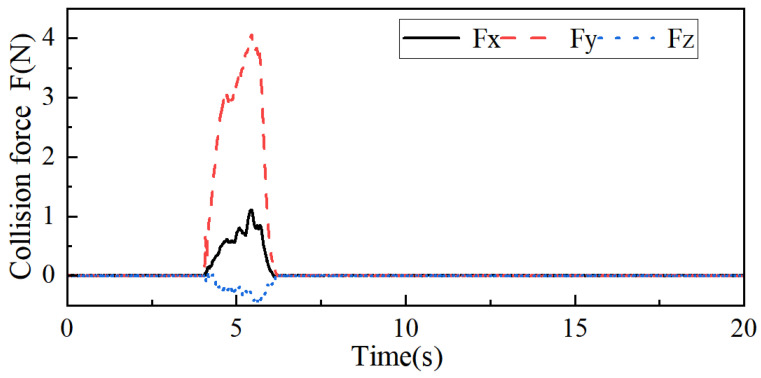
Collision force information F.

**Figure 25:**
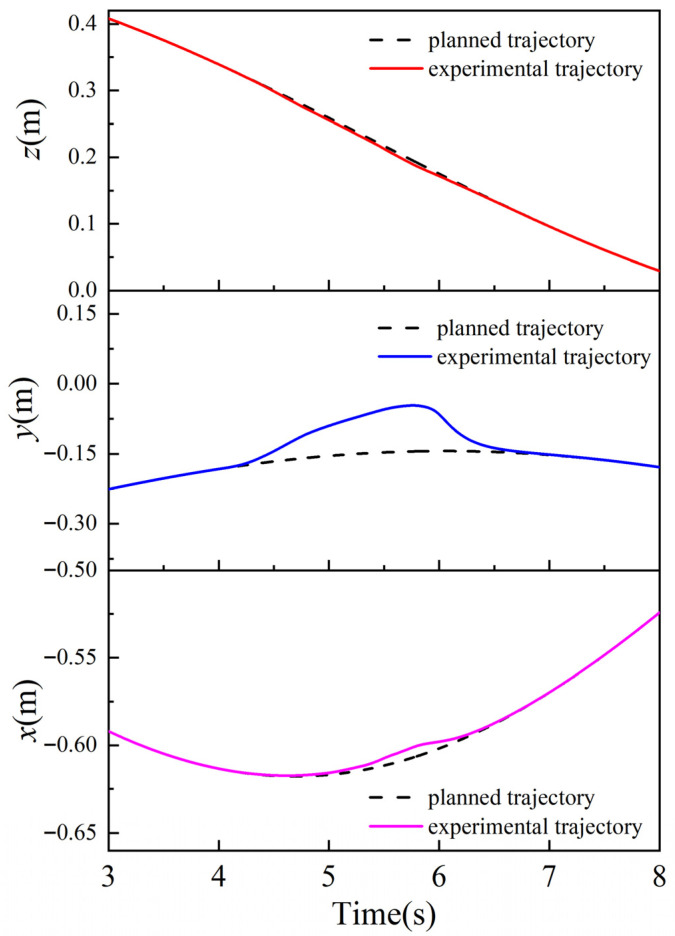
Robot end-avoidance results.

**Figure 26:**
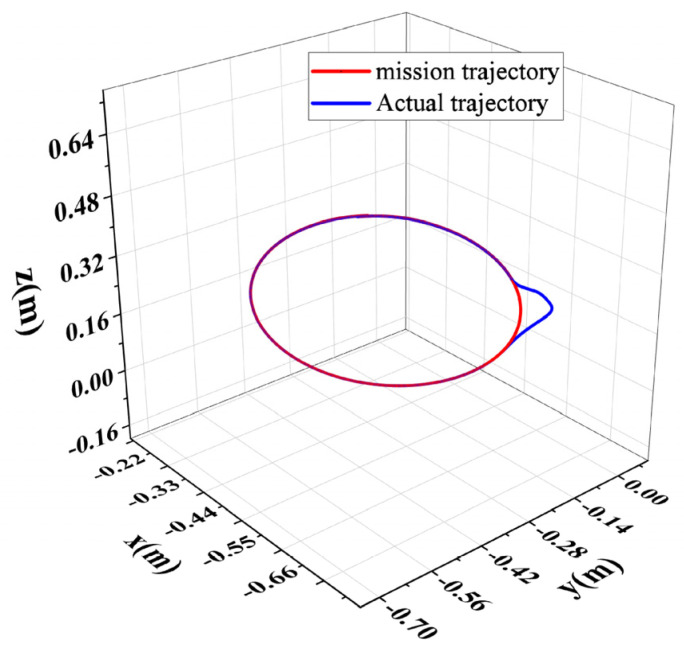
Robot end-of-task trajectory and actual trajectory.

Information F, about the collision force on the robot end in the x-, y-, and z-axis directions, detected by a six-dimensional force sensor, is shown in Figure 24; the position of the robot end when the robot performs a collision response is shown in Figure 25; and the avoidance trajectory generated based on the human–robot collaborative collision avoidance control algorithm is shown in Figure 26. As can be seen from Figure 24 and Figure 26, the maximum collision forces acting on the x-, y-, and z-axes of the robot end during the collision-response process are $F_{xmax}$ = 1.12 N, $F_{ymax}$ = 4.06 N, and $F_{zmax}$ = −0.44 N, respectively, and under the effect of the above collision forces, the maximum positional deviation of the robot end in the x-axis is 6.94 mm, in the y-axis 98.75 mm, and in the z-axis 5.43 mm, and the robot can track to the original task trajectory within 1 s after the collision disappears. When running on an actual industrial PC at 1 kHz, the algorithm’s average single iteration time is only 0.12 ms, with a worst-case of 0.18 ms and CPU usage < 3%. This demonstrates the algorithm’s high real-time performance, making it easily deployable on existing controllers.

#### 3.5.2. Collaborative Robot Body Collision-Response Experiment

**Figure 27:**
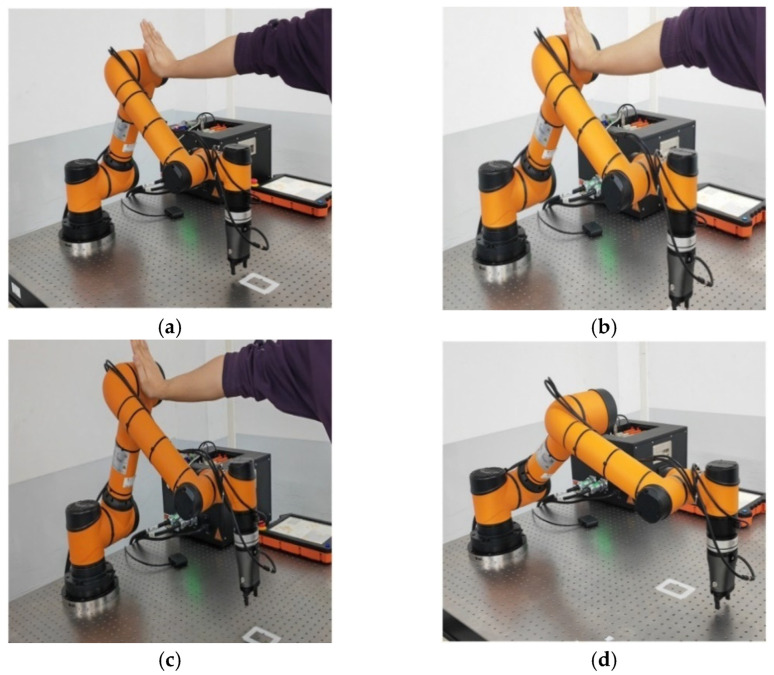
Robot arm collision-response process. (**a**) Pre-collision. (**b**) Collision. (**c**) Collision response. (**d**) Resumption of mission trajectory.

The AUBO-i5 robot arm collision-response experiment is also based on the predefined square trajectory presented in Section 4, and the other experimental parameters were the same as in the simulation experiment. The collision force information in the robot joint space is provided by the integrated joint module detection method. Volunteers applied an external force to the big arm of the AUBO-i5 robot by hand to simulate the collision-response experiment of the robot body. The collision-response process of the AUBO-i5 robot is shown in Figure 27.

**Figure 28:**
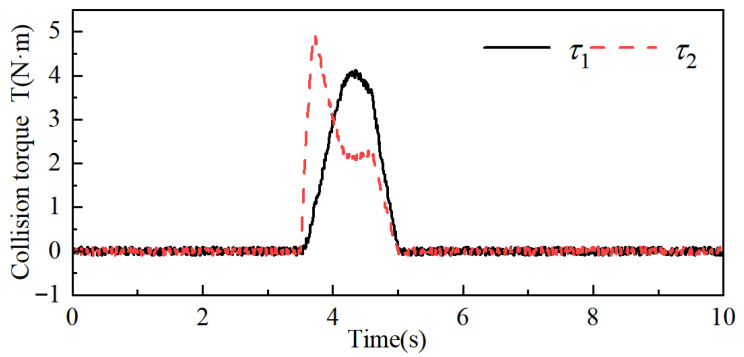
Crash moment information T.

**Figure 29:**
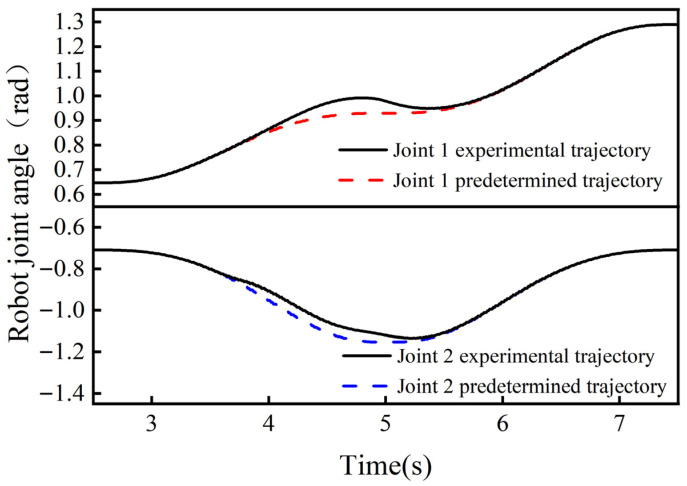
Collision avoidance results for robot joints 1 and 2.

When an external collision force is applied to the robot’s big arm, the collision moments of robot joints 1 and 2 are generated, and the collision moments of the other joints are zero. The collision moments of robot joints 1 and 2 are obtained by the joint moment detection method proposed in Section 3, as shown in Figure 28, and the avoidance angles generated by robot joints 1 and 2 are shown in Figure 29. From Figure 28 and Figure 29, it can be seen that the maximum collision moments acting on robot joints 1 and 2 during the collision response of the robot’s big arm are t1 = 4.13, t2 = 1, and under the effect of the above collision moments, the maximum angular deflection of joint 1 is 0.0636 rad, and the maximum angular deflection of joint 2 is 0.0521 rad, and the robot is able to track the robot to the original task trajectory within 0.85 s after the collision disappears. The results of this experiment show that the robot body can also show good flexibility under the effect of smaller collision moments to achieve the purpose of safe control of human–robot collaboration.

## 4. Discussion

The findings of this study align with and extend previous research on robot collision response, addressing key limitations of existing strategies. Unlike emergency braking and reflex control, which often cause task interruptions, the proposed adaptive admittance control achieves both compliant collision response and seamless trajectory recovery, which resonates with the core demand for continuous operation in human–robot collaboration emphasized by Zhou and KIM. The superior performance in reducing response time and increasing avoidance displacement compared to fixed-parameter and variable stiffness admittance control validates the working hypothesis that the synergistic adjustment of stiffness and damping parameters outperforms single-parameter tuning. These results underscore the potential of adaptive admittance control to enhance safety and efficiency in dynamic collaborative scenarios such as industrial assembly and healthcare assistance.

The adaptive admittance control-based method proposed in this research forms a distinct complement to existing mainstream schemes in terms of theoretical foundation. Although neural network-based collision detection methods possess robust data-driven perception capabilities, their “black-box” decision-making nature leads to limitations in the provability of their safety and their physical interpretability. Reinforcement learning-based adaptive methods hold potential in complex strategy optimization, yet they face theoretical challenges such as online learning risks, difficulty in providing a priori stability guarantees, and high sample complexity in safety-critical scenarios. While impedance control systems with disturbance observers exhibit strong robustness against model uncertainties, the proposed method aims to more directly and optimally address the core contradiction between “compliance” and “task continuity” through the coordinated adaptation of stiffness and damping, rather than merely focusing on disturbance suppression. In summary, this study provides a solution with a solid theoretical basis, high computational efficiency, and ease of deployment.

## 5. Conclusions

First, a full-dimensional force feedback fusion collision detection system was proposed based on the collision conditions in the human–robot collaboration environment, which integrates a base six-dimensional force sensor, an integrated joint module, and a six-dimensional force sensor for the wrist. Second, a Simulink simulation model of admittance control was established according to the principle of admittance control, and the influence of admittance parameters on the system performance was analyzed using the control variable method. The study found that the magnitudes of the stiffness and damping parameters affect the avoidance displacement and response time during collision response. Therefore, an adaptive admittance control collision-response algorithm was proposed, and a collision simulation model of the AUBO-i5 collaborative robot was constructed. Through simulation experiments on end-effector collisions and body collisions of the robot and comparisons with existing admittance control algorithms, the results showed that in the end-effector collision experiments, the maximum positional offsets on the x-, y-, and z-axes were improved by 17.46%, 8.89%, and 13.09%, respectively, and the response times were reduced by 7.94%, 14.89%, and 8.21%, respectively. In the body collision experiments, the avoidance angles of Joint 1 were improved by 39.49% and 234.95%, and the response times were reduced by 7.16% and 14.78%, respectively. The experimental results verified the effectiveness of the proposed adaptive admittance collision-response algorithm. Finally, a collision-response experimental platform based on the AUBO-i5 collaborative robot was built, and simulated collision experiments were conducted on the robot’s end-effector and body, respectively. The experimental results demonstrated that the collision detection system proposed in this paper can promptly detect and feedback collision force information when a collision occurs. The adaptive admittance control collision-response algorithm exhibited good compliance during the collision-response process, and the robot was able to track back to the original task trajectory within 1 s after the collision ended, achieving the goals of reducing collision forces and improving work efficiency. Future research can explore integrating sensor fusion technologies to improve the accuracy of collision force/moment estimation or combining this responder with a perception module featuring strong generalization capabilities (such as a neural network detector). Additionally, an integrated digital twin model of robots and working scenarios could be constructed, multi-physics field coupling models could be further integrated with data-driven calibration to enhance the model’s adaptability to dynamic environments and prediction accuracy, and multi-modal perception fusion algorithms could be optimized to improve the real-time performance and robustness of collision detection in complex scenarios.

## Data Availability

The original contributions presented in this study are included in the article. Further inquiries can be directed to the corresponding authors.
